# Changes of soil bacterial community composition and functional groups in different altitude gradients of *Potentilla fruticosa* shrub in eastern Qinghai-Tibet Plateau

**DOI:** 10.3389/fpls.2025.1539945

**Published:** 2025-10-20

**Authors:** Lele Xie, Yushou Ma, Yanglong Wang, Yuan Ma, Ying Liu

**Affiliations:** ^1^ Qinghai Academy of Animal and Veterinary Science, Academy of Animal and Veterinary Science, Qinghai University, Xinin, Qinghai, China; ^2^ Qinghai University Qinghai Provincial Key Laboratory of Adaptive Management on Alpine Grassland, Xining, Qinghai, China

**Keywords:** alpine ecosystem, bacterial community, different altitudes, co-occurrence network, soil properties

## Abstract

**Introduction:**

Soil properties and extracellular enzyme activities are the key factors affecting microbial communities at different altitudes. It is very important to understand their distribution patterns along the elevation gradient for predicting the function of alpine ecosystems in response to climate change, especially in alpine shrub ecosystems, which are rarely studied.

**Methods:**

In eastern Qinghai-Tibet Plateau Set up four sample plots with different altitudes (3400,3700,4000,4300 and 4300 m), Illumina I6S gene sequencing was used to analyze the changing law of soil bacterial community, and its diversity and influencing factors were discussed by combining correlation and redundancy analysis.

**Results:**

The results showed that the contents of soil total carbon, total nitrogen, water content, microbial biomass carbon, nitrogen, phosphorus and alkaline phosphatase were the highest, but the bacterial diversity was the lowest (Shannon and Pielou index); Dominant bacteria are different at different altitude gradients. Acidobacteriota is the most abundant at 3700, 4000 and 4300 m above sea level, while Verrucomicrobiota is the most abundant at 3700m above sea level; In addition, PCoA analysis showed that the community structure of soil bacteria changed significantly, with similar structures at 3700 , 4000 and 4300 m above sea level; Redundancy analysis (RDA) showed that soil properties (TC and BD) and enzyme activities (AG, BG and CBH) were the main factors affecting the bacterial community, while soil properties (TN), microbial biomass (MBC and MBN) and enzyme activities (AG and BG) all had significant effects on the functional groups of soil bacteria.

**Conclusion:**

To sum up, these results show that soil physical and chemical properties, microbial biomass, enzyme activities and bacterial communities have different responses to different altitude gradients.These studies provide a new perspective for us to deeply understand the driving factors of soil bacterial community variation along the elevation gradient. It is helpful to strengthen our awareness of the protection of Potentilla fruticosa shrub, and also provide basic information for the study of alpine ecosystem in this area.

## 1 Introduction

Soil microorganisms and enzymes are important components of soil and play a significant role in the ecosystem (Shu et al., 2023). They are not only regulators of ecosystem functions but also the main drivers of nutrient transformation and material cycling in the ecosystem (Wang et al., 2025). The main groups of soil microorganisms include bacteria, fungi, actinomycetes, and protozoa. These microorganisms can secrete enzymes such as alkaline phosphatase, β-1,4-glucosidase, cellulase, amidase, and nitrogenase (Arunrat et al., 2024). Through decomposition, they can mineralize soil organic matter, including remains of animals and plants, and release nutrients into the surrounding soil, playing an important role in the normal growth of plants and the stability of ecosystem structure and function (Wyszkowska et al., 2019). As the main group of soil microorganisms, soil bacteria have complex ecological functions and promote the decomposition of soil organic matter by participating in the cycle and metabolism of soil nutrients (Yan et al., 2023). Soil moisture, organic carbon, and total nitrogen are generally important environmental factors that influence the composition of soil bacterial communities (Pang et al., 2023). In addition, changes in the environment of soil ecological processes can lead to significant changes in soil bacterial communities at the regional scale (Crowther et al., 2019). Especially in alpine shrub ecosystems, the distribution pattern of soil bacterial communities along altitudinal gradients has always been a hot topic in ecological research (Zhu et al., 2025). Therefore, analyzing the soil bacterial communities in the *P*.fruticosa shrubs of the Yellow River source area on the Qinghai-Tibet Plateau is of great significance for the biodiversity conservation and ecological management of alpine ecosystems.

In the alpine shrub ecosystem, there are significant differences in climate, plant and soil characteristics in a short spatial distance (Bayranvand et al., 2021; Zhao et al., 2025). Altitude gradient is considered as a powerful “natural laboratory” to study the response and feedback of soil function to climate change (Feng et al., 2021). Especially the altitude model to detect soil microbial community and extracellular enzyme activity, because the climate and biological properties change dramatically in a small spatial scale (Klimek et al., 2020). The variation patterns of soil bacterial community composition, biomass and soil extracellular enzyme activity along altitude gradient were studied extensively (Ren et al., 2018). For example, some studies show that microbial biomass increases, decreases or has no obvious trend with the elevation (Chang et al., 2016; Klimek et al., 2020). In Nyainqentanglha Mountain (Si et al., 2014) and Italian Alps (Margesin et al., 2014), it was found that soil extracellular enzyme activity decreased along the elevation gradient, while in south-central Chile (Reyes et al., 2011) and Wuyishan (Jin et al., 2011), it was found that soil enzyme activity increased with altitude. A study of Kohala volcano in Hawaii by Peay et al. (2017) found that the bacterial diversity showed a hump-shaped upward trend. In a study in Mount Fuji, Singh et al. (2012) found that the diversity of soil bacteria and the abundance of Acinetobacter decreased monotonously with the increase of altitude gradient. Cui et al. (2019) observed the different changes of α-diversity of soil bacteria in Qinghai-Tibet Plateau at high altitude gradient, and the results showed that Shannon-Wiener diversity of bacteria first increased and then decreased. The above research found that the research results of bacterial community and extracellular enzyme activity were inconsistent, and the consensus was still difficult to reach. This shows that the influencing factors are not only the temperature fluctuation caused by altitude rise, but these contradictory observations may be attributed to the interaction of various abiotic and biological factors on bacterial communities and extracellular enzyme activities (Chang et al., 2016; Siles et al., 2017; Zhao et al., 2025). Because this area is very sensitive to climate change, there is still a lack of synchronous understanding of the changes of soil bacterial community and extracellular enzyme activity along the elevation gradient, which needs to be solved urgently (Fan et al., 2021).

At present, the research on the response of soil bacteria to altitude gradient mainly focuses on large-scale areas and different vegetation types (Yuan et al., 2014). However, the mechanism behind the small-scale changes of soil bacterial community structure in the same vegetation type, especially in the *P. fruticosa* shrub, is limited. In view of that fact that the response of soil microbial community structure to altitude gradient change has become an important part of predict the response of ecosystem to environmental change (Cui et al., 2019), It is necessary to study the response mode and driving factors of soil microorganisms in shrub communities with the same dominant species to altitude gradient changes, so as to improve people’s awareness. *P. fruticosa* shrub is widely distributed on shady slopes, semi-shady slopes and diluvial fans in mountainous areas at an altitude of 3 200~4 500 m, and is an important shrub species in alpine ecosystem, which plays an important role in maintaining the function of alpine meadow ecosystem in Qinghai-Tibet Plateau (Li et al., 2010).

Therefore, in this study,*P. fruticosa* shrub investigated along different altitudes in alpine shrub ecosystem was taken as the research object, and Illumina MiSeq technique was used to analyze the composition of soil bacterial community and extracellular enzyme activities related to C, N and P acquisition in the meadow of *P.fruticosa* shrub at altitude. By studying the important correlation between soil bacterial community and extracellular enzyme activities and soil variables, Characterizing changes in soil bacterial communities and extracellular enzyme activities along altitude, and identifying possible drivers of changes in soil bacterial communities and extracellular enzyme activities along altitude are of great significance in guiding ecological restoration and regeneration of Source region of the Yellow River in the eastern part of the Qinghai-Tibetan Plateau and realize the sustainable management of shrub meadow. We assume that: (i) at high altitude (3700 m), due to the combined influence of soil variables, soil microbial biomass and extracellular enzyme activities related to N and P cycles increased significantly, while extracellular enzyme activities related to carbon cycle decreased significantly; (ii) α diversity and composition of soil bacterial communities will change with altitude. (iii) Soil factor (TC) will be an important factor affecting the bacterial community, because it can affect the soil microbial community by driving C and nutrients into the soil microorganisms during the nutrient flow.

## 2 Materials and methods

### 2.1 Study area

Golog Tibetan Autonomous Prefecture in Qinghai Province is located in the hinterland of the Qinghai-Tibet Plateau (96°54′∼101°51′E,32°31′∼35°37′N), at the Source region of the Yellow River, with an average elevation of over 4,200 m, with an annual average temperature of-0.4°C∼3.7°C, annual precipitation of 400-760 mm and annual sunshine hours of 1,988. The vegetation type is alpine meadow and the soil is mainly alpine shrub meadow soil (Xie L. L. et al., 2024). Common herbs are Cyperaceae, such as*Carex capillifolia*, *Carex alatauensis* and *Carex atrofusca Schkuhr*; Gramineae includes *Elymus nutans Griseb*, *Poa pratensis* L. and *Deschampsia cespitosa*; Miscellaneous grasses include *Bistorta vivipara*, *Pedicularis kansuensis Maxim*, and *Anaphalis lactea Maxim*.

### 2.2 Experimental design and sample collection

**Figure 1:**
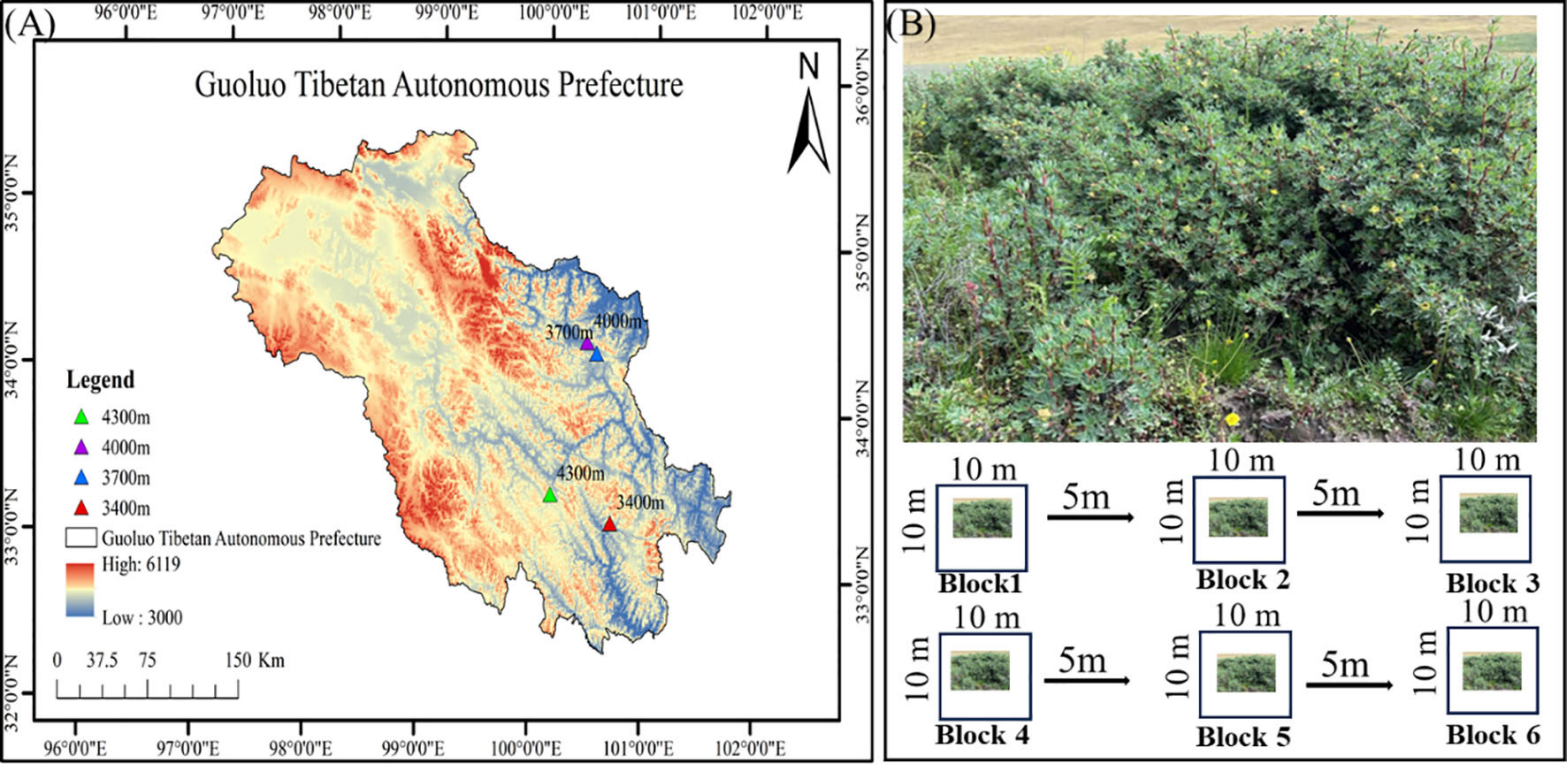
Distribution **(A)** and sampling method **(B)** of four experimental sites in Guoluo Tibetan Autonomous Prefecture.

In the study area, the experimental sites were selected according to the following principles: (1) The slope and aspect were consistent, and *P. fruticosa* was the dominant shrub species in the sample plot. (2) In all the experimental sites, the growth status of *P. fruticosa* shrub is similar; (3) The environmental conditions of each experimental site are relatively consistent, that is, the topography and soil types are basically the same; (4) Based on the above principles, this study selected four experimental sites (3400, 3700, 4000, and 4300 m) with a span of 300 m. The detailed distribution is shown in **Figure 1**.

The sampling time of this study is August, 2023, the peak season of forage growth, in the altitude range of 3400 -4300 m, taking 300 m as an altitude span to illustrate the difference of different altitude gradients. In order to reduce the spatial heterogeneity, we set up 6 repeated plots in each experimental site, and the interval between each plot is 10 m. Therefore, we have a total of 24 plots (4 experimental sites and 6 repeated plots). Field quadrat survey was used to record the species composition and coverage of each plot, and the latitude, longitude and altitude of each experimental point were recorded by GPS. A total of 24 soil samples were collected in this study (Bai et al., 2020).

At each elevation gradient, six 10m×10m shrub quadrats were established. Within each quadrat, five sampling points were set up using the five-point sampling method. After removing surface litter and stones from each sampling point, soil samples were collected from the 0-20 cm depth using a soil auger with a diameter of 3.5 cm. The soil samples from the five sampling points within each quadrat were thoroughly mixed to form a composite soil sample. Therefore, a total of 24 soil samples were collected in this study (4 elevation gradients × 6 replicates). Pass the soil sample through a 2 mm sieve to remove roots and stones, and then the soil samples are immediately sent back to the cooler in the laboratory. The soil sample is divided into two parts, which are used to determine the chemical properties and microorganisms existing in the soil. A portion soil samples are naturally air-dried indoors to determine soil physical and chemical properties and other indicators. The other part was stored at -80°C for Qualcomm gene sequencing (Zhou et al., 2024).

### 2.3 Analysis of soil nutrients and enzyme activities

The contents of total nitrogen (TC) and total nitrogen (TN) in soil were determined by C and N analyzer (Elementar, Langenselbold, Germany);Total phosphorus (TP) in soil was determined by molybdenum antimony colorimetric method and flow analyzer (Proxima, AMS Alliance, Paris, France); pH measurement: Measure the soil pH from the sample containing the ratio of soil (air-dried) to water of 1:5; Soil moisture content (SM): measure the soil moisture content after drying in an oven at 105°C for 48 h to constant weight; BD: The density (BD) of soil samples was measured by circular knife method and dried at 105°C for 24 hours. Soil microbial biomass carbon (MBC) and microbial biomass nitrogen (MBN) were determined by chloroform steaming-K_2_SO_4_ leaching method, and soil microbial biomass phosphorus (MBP) was determined by chloroform steaming-NaHCO_3_ leaching method (Zhao et al., 2023).

Six important enzymes in soil carbon, nitrogen and phosphorus cycling were selected to determine their activities, among which β-D-glucosidase (BG), cellulose-degrading cellobiohydrolase (CBH) and α-1,4-glucosidase (AG) were C-harvesting enzymes (Cenini et al., 2015). β-1,4-N-acetylglucosamine glucosidase (NAG) and Leucine aminopeptidase (LAP) are common acquisition enzymes (Salazar et al., 2020). Alkaline phosphatase (ALP) is p-acquiring enzyme (Delgado-Baquerizo et al., 2017). According to the method of Saiya-Cork et al. (2002) the activity of extracellular enzymes in soil was determined: firstly, 1g of soil was weighed and put into a jar filled with 125ml acetic acid buffer with pH 5.05 mmol/L, and stirred for 5min with a magnetic stirrer to homogenize the soil and solution; Then use a pipette to take 200μL of suspension. Add it to a 96-well plate, put it in the dark at 20°C for 4 hours, and then add 10μL 1 mol/L NaOH solution; Finally, the soil enzyme activity was determined by a multifunctional enzyme marker (Synergy H, USA) (Saiya-Cork et al., 2002).

### 2.4 16S amplicon sequencing method and analysis

DNA was extracted from 0.5g of soil sample using the Hipure Soil DNA Kit (Magen, Guangzhou, China). 2µL of the DNA sample was taken and the OD value of the nucleic acid was measured using a NanoDrop 2000 micro-spectrophotometer to detect the purity of the nucleic acid. Agarose (1% agarose) gel electrophoresis was used to detect the integrity of the nucleic acid sample and the degree of protein contamination. 2µL of the DNA sample was taken and the DNA concentration of each sample was detected using Qubit fluorescence quantification (Qubit 3.0, Thermo Fisher, USA), and the total DNA amount of the sample was calculated based on the concentration quantification results. Specific primers with barcodes were used to amplify the V4 region of the bacterial 16S rRNA, and the primers used were 515 F: (GTGYCAGCMGCCGCGGTAA) and 806 R (GGACTACNVGGGTWTCTAAT) (Zhao et al., 2023).

After obtaining the raw sequence (Raw reads), low-quality sequences (reads) are filtered, followed by assembly and re-filtering to obtain representative sequences of operational taxonomic units (OTUs). Uparse software is used to cluster all effective sequences of all samples, and sequences are clustered into OTUs with 97% consistency, and the absolute abundance and relative information of each OTU in each sample are calculated (Li et al., 2016). Select all OTUs with an average abundance greater than 1 in the comparative groups (i.e., high-abundance union OTUs) for Venn analysis. During the construction of OTUs, Uparse will select representative sequences (the Tag sequence with the highest abundance in the OTUs). These representative sequences are then used with the Naïve Bayesian assignment algorithm of the RDP Classifier to annotate species against the Silva database (Edgar, 2013), with a confidence threshold set between 0.8 and 1.0 (Xie L. L. et al., 2024). Based on the species annotation information of OTUs, count the number of Tags sequences for each sample at the phylum level. Based on the counting statistics of OTUs, analyze the differences in soil bacterial communities using the Bray-Curtis distance coefficient. Use Faprotax to predict the ecological functions of soil bacteria.

### 2.5 Statistical analysis

Excel 2021 is used to sort out the data, and then Kolmogorov-Smirnov is used to test the normality of the data to determine whether it meets the normality hypothesis of the subsequent statistical test. SPSS 24 (IBM Corp., Armonk, NY, USA) was used to analyze the diversity index (Simpson, Shannon, Pielou and good_coverage index) by one-way analysis of variance (one-way ANOVA; α=0.05) to determine the significance between different altitude gradients, and the least significant difference method (LSD) is used for multiple comparisons. All data in the table are average standard error. Principal Coordinate Analysis (PCoA) is a dimensionality reduction analysis of microbial community based on Unweight unifrac distance, which evaluates the degree of explanation of bacterial community structure on each coordinate axis by percentage. The relationship between bacterial colony structure and soil physical and chemical properties was obtained by redundancy analysis,RDA) of Canoco 5.0 software package, and was plotted by Origin 2021. Linear discriminant analysis(LDA) effect size (Lefse) method is used to determine the microbial groups with significant differences among soil samples, and the unique microbial groups are detected with LDA threshold of 2.5 and significance less than 0.05, and all samples are compared (Zhang and Laanbroek, 2020). One-way similarity analysis (ANOSIM) based on unweighted UniFrac distance was used to test whether the difference of β diversity between treatments was significantly higher than that within groups. The relationship between α and β-diversity and bacterial community was discussed by Mantel test. The correlation network diagram between microbial community at the door level and soil physical and chemical properties was constructed by Spearman correlation coefficient using vegan software package. Then, a molecular ecological network including all samples was created, and the abundance of OTU was normalized by using the Hmisc package of R, and the Spearman correlation among parameters was estimated. Then, the molecular network was constructed by using the RMT model. Subsequently, Gephi 9.2 was used to visualize the network and calculate the parameters, including the number of nodes and edges, the average degree (avgK), the average path length (GD) and the average clustering coefficient (avgCC), to determine the interaction of bacteria between different altitude gradients (Barberán et al., 2012). According to OTU classification information and FAPROTAX database, the function of soil bacterial community was predicted.

## 3 Results

### 3.1 Soil properties, microbial biomass and enzyme activities at different altitude gradients

The results show that altitude has significant effects on soil total carbon, total nitrogen, soil bulk density, microbial biomass carbon, microbial biomass nitrogen and microbial biomass phosphorus (**Table 1**). Soil total carbon and total nitrogen first increased and then decreased with the elevation, all of which were significantly higher than 3400, 4000 and 4300 m. With the elevation, the soil bulk density increased first, then decreased, and then increased, and the soil bulk density content reached the maximum at 3400 m. The biomass carbon, nitrogen, and phosphorus of microorganisms first increased, then decreased, and then increased again along the altitude gradient. Specifically, within the range of 3400-4300m, the content of all three reached their peak at 3700m (MBP: 21.50 ± 4.91mg/kg; MBC: 2334.49 ± 268.49 mg/kg; MBN: 162.16 ± 29.19 mg/kg), then significantly decreased at 4000m, and showed an increasing trend again at 4300m. Compared to the microbial biomass at 4000m, phosphorus, carbon, and nitrogen increased by 10.27 mg/kg, 478.05 mg/kg, and 67.49 mg/kg, respectively, at 4300m.

**Table 1:** Changes of soil nutrients and microbial biomass in different altitude gradients.

Altitude	3400	3700	4000	4300
TC	41.57 ± 0.50c	85.28 ± 1.20a	48.90 ± 1.23b	37.52 ± 0.96b
TN	4.07 ± 0.70b	7.36 ± 0.56a	4.51 ± 0.11b	3.77 ± 0.21b
TP	0.50 ± 0.04a	0.54 ± 0.06a	0.57 ± 0.07a	0.57 ± 0.05a
pH	6.99 ± 0.13a	6.76 ± 0.17a	7.09 ± 0.07a	6.97 ± 0.15a
SM	0.64 ± 0.03a	0.65 ± 0.04a	0.58 ± 0.03a	0.59 ± 0.04a
BD	0.82 ± 0.02a	0.34 ± 0.02c	0.73 ± 0.06ab	0.65 ± 0.05b
MBP	16.84 ± 3.50a	21.50 ± 4.91a	7.92 ± 1.70b	18.19 ± 5.82a
MBC	1496.19 ± 97.30c	2334.49 ± 268.49a	1633.17 ± 115.36bc	2111.22 ± 130.02ab
MBN	108.43 ± 10.88ab	162.16 ± 29.19a	53.59 ± 8.51b	121.08 ± 31.27ab

3400, at an altitude of 3400 m; 3700, at an altitude of 3700 m; 4000, at an altitude of 4000 m,4300, and 4300 m.TC, TN, TP, pH, SM, BD, MBP, MBC and MBN soil total carbon, total nitrogen, total phosphorus, pH, soil water content, soil bulk density, microbial biomass phosphorus; microbial biomass carbon; microbial biomass nitrogen respectively, Values are the means ± SE (n=6). Lowercase letters indicate significant differences among different altitude gradients (*P* < 0.05).

**Figure 2:**
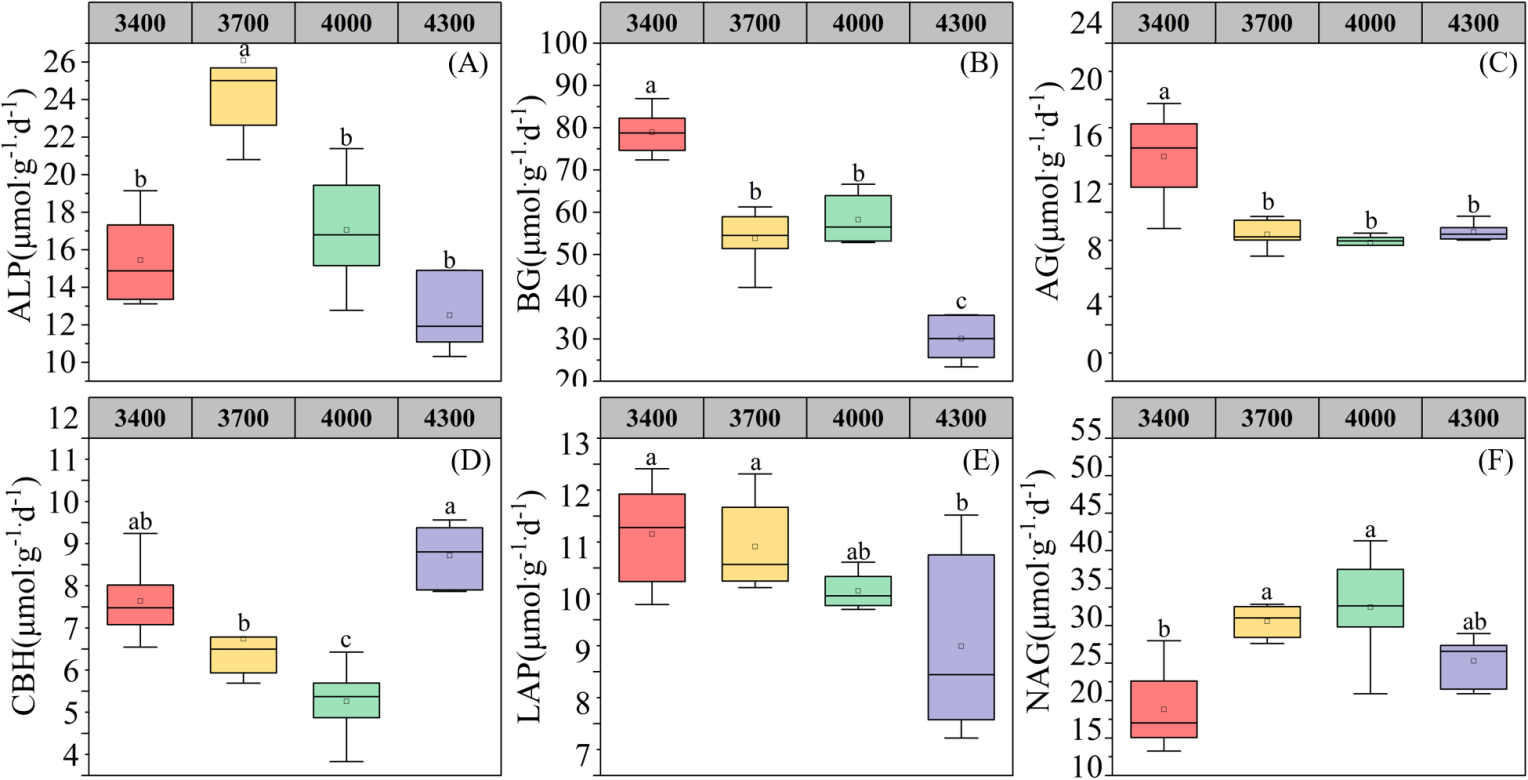
Changes of soil enzyme activities at different altitudes. alkaline phosphatase **(A)**;BG, β-1,4-glucosidase **(B)**; AG,α-1,4-glucosidase **(C)**;CBH, β-D-cellobiosidase **(D)**; LAP, L-leucine aminopeptidase **(E)**; NAG, β-1,4-N-acetylglucosaminidase **(F)**. Different lowercase letters indicate significant differences at the 0.05 confidence level.

As shown in **Figure 2**, the single factor analysis of variance shows that altitude significantly affects the enzyme activities related to soil carbon, nitrogen and phosphorus cycling (**Figure 2**). With the increase of altitude, the activities of enzymes related to soil phosphorus cycle first increased and then decreased, reaching the highest at 3700 m (**Figure 2A**). Altitude significantly affected the enzyme activities related to soil carbon cycle (β-1,4-glucosidase, α-1,4-glucosidase and β-D-cellobiohydrolase). Among them, β-1,4-glucosidase and α-1,4-glucosidase decreased with the elevation (**Figures 2B, C**), while β-D-cellobiohydrolase increased first and then decreased with the elevation (**Figure 2D**). L-leucine aminopeptidase related to soil nitrogen cycle decreased with the elevation, reaching the lowest at 4300 m (**Figure 2E**), while β-1,4-N-acetylglucosaminidase first increased and then decreased with the elevation, reaching the highest at 4000 m (**Figure 2F**).

### 3.2 α diversity of bacterial communities under different altitude gradients

**Figure 3:**
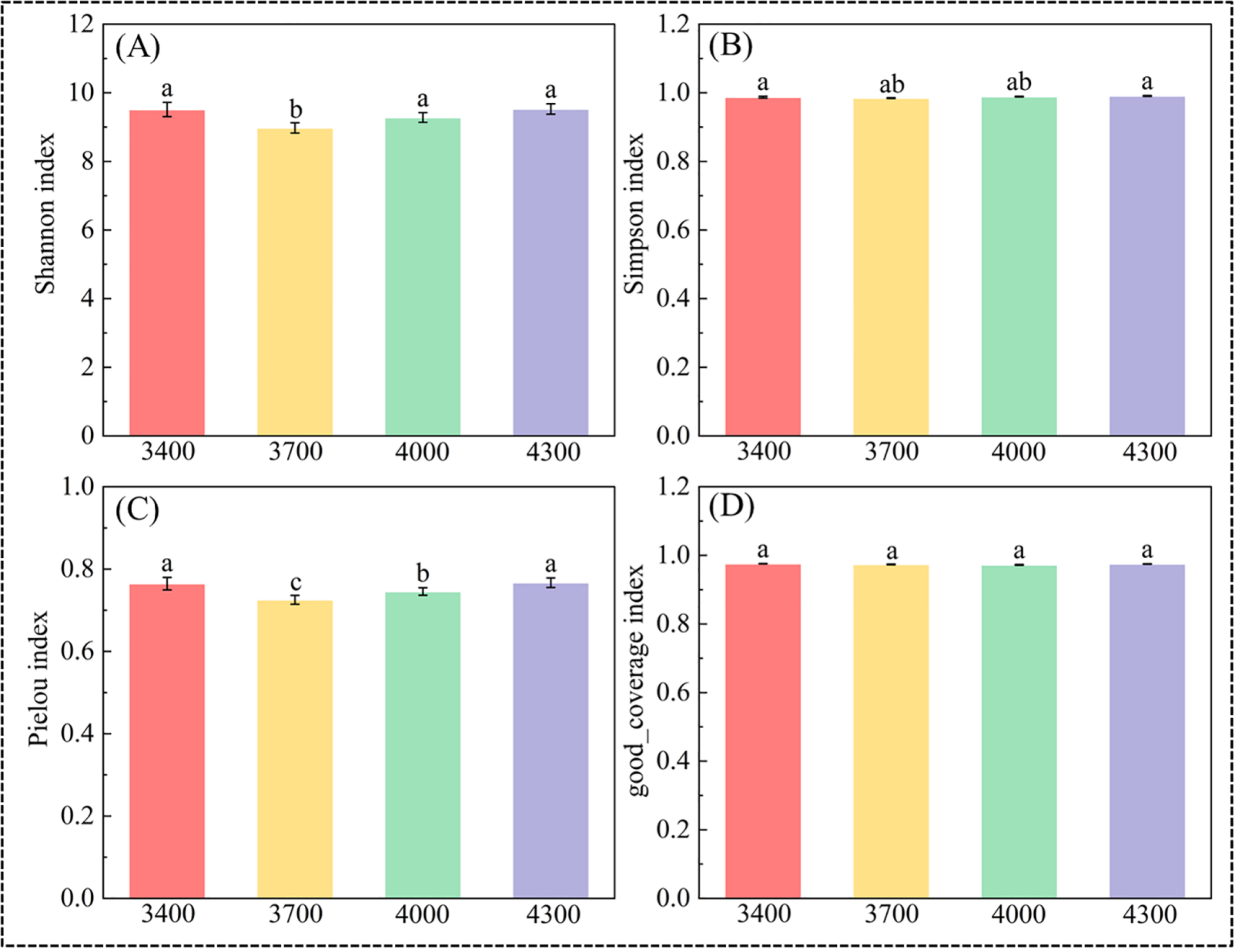
Diversity and richness index of bacteria in different altitude gradients. Shannon index **(A)**, Simpson index **(B)**, Pielou index **(C)**, and good coverage index **(D)**. Different lowercase letters indicate significant differences at the 0.05 confidence level.

As shown in **Figure 3**, there are significant differences in bacterial α diversity among different altitude gradients (**Figure 3**). With the increase of altitude, the Shannon index first increased and then decreased, and the Shannon index reached its peak at 4300 m (9.52) (**Figure 3A**). However, the difference of good_coverage index between different altitudes is not significant (**Figure 3D**). Simpson index and Pielou index first decreased and then increased with the elevation, and both reached the peak at 4300 m, which were 0.99 and 0.76 respectively (**Figures 3B, C**).

### 3.3 Composition and structure of bacterial community under different altitude gradients

**Figure 4:**
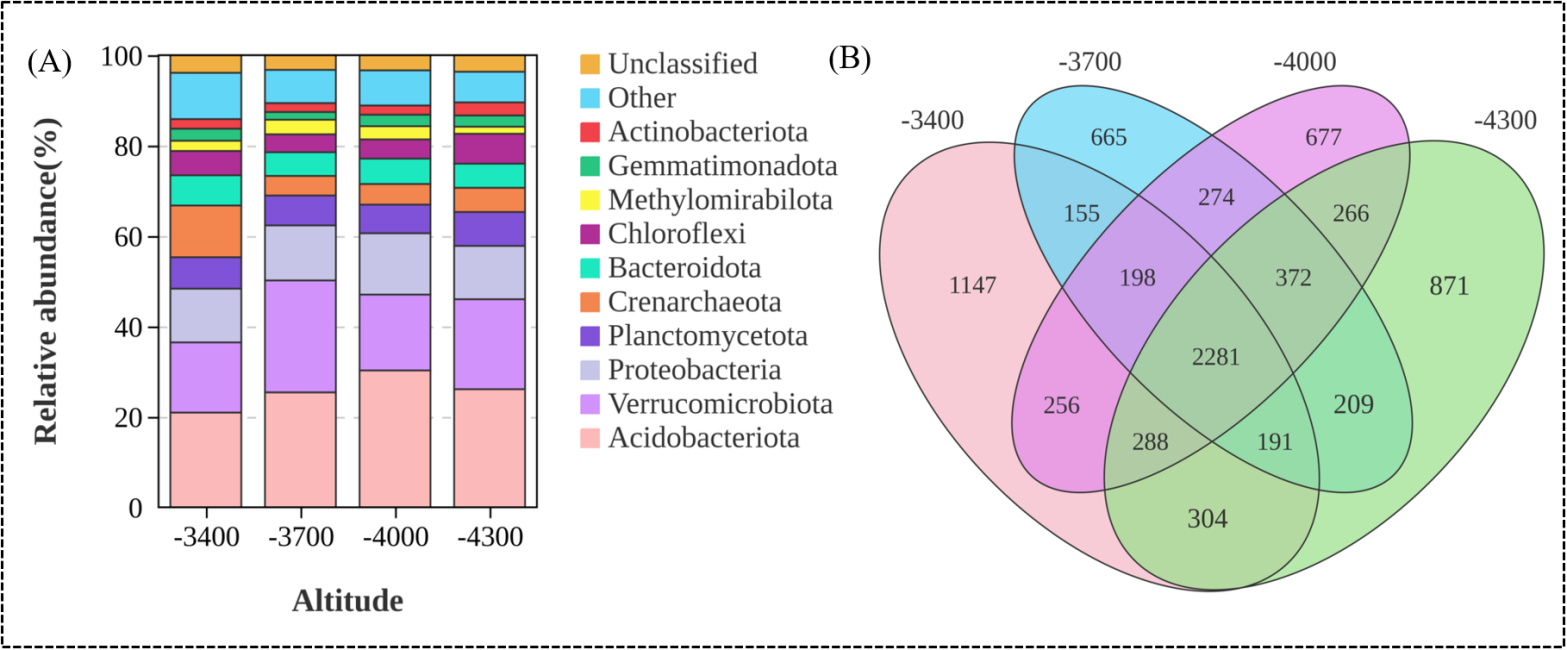
Composition of soil bacterial community at the door level **(A)**, Venn analysis of bacteria OTU at different altitude gradients **(B)**.

At the phylum level (**Figure 4A**), the dominant groups of soil bacteria in different altitude gradients are mainly Acidobacteriota, Verrucomicrobiota, Proteobacteria and Planctomycetota, with abundance of 25.69%, 19.24%, 12.36% and 6.85% respectively. The relative abundance of bacteria at different phylum levels varied significantly with altitude gradient (*P*<0.05). With the elevation, Acidobacteriota first increased and then decreased, reaching the maximum relative abundance (30.27%) at 4000 m, while Verrucomicrobiota showed a downward trend with the elevation gradient, and the relative abundance was the lowest (16.77%) at 4000 m. Proteobacteria first increased and then decreased with the elevation, with the highest relative abundance (13.57%) at 4000 m, while Planctomycetota showed an upward trend with the elevation, with the highest relative abundance (7.49%) at 4300m (**Figure 4A**). Venn shows that there are 35 phylum between different altitude gradients, and the total number of bacteria OTUs under different altitude gradients is 8144 (**Figure 4B**).

A total of 2281 operational taxonomic units (OTUs) were identified. The number of OTUs at different altitudes was 4820 (3400 m), 4345 (3700 m), 4612 (4000 m), and 4782 (4300 m), respectively. Correspondingly, the number of unique bacterial OTUs at these altitudes was 1147 (3400 m), 665 (3700 m), 677 (4000 m), and 871 (4300 m), respectively.

**Figure 5:**
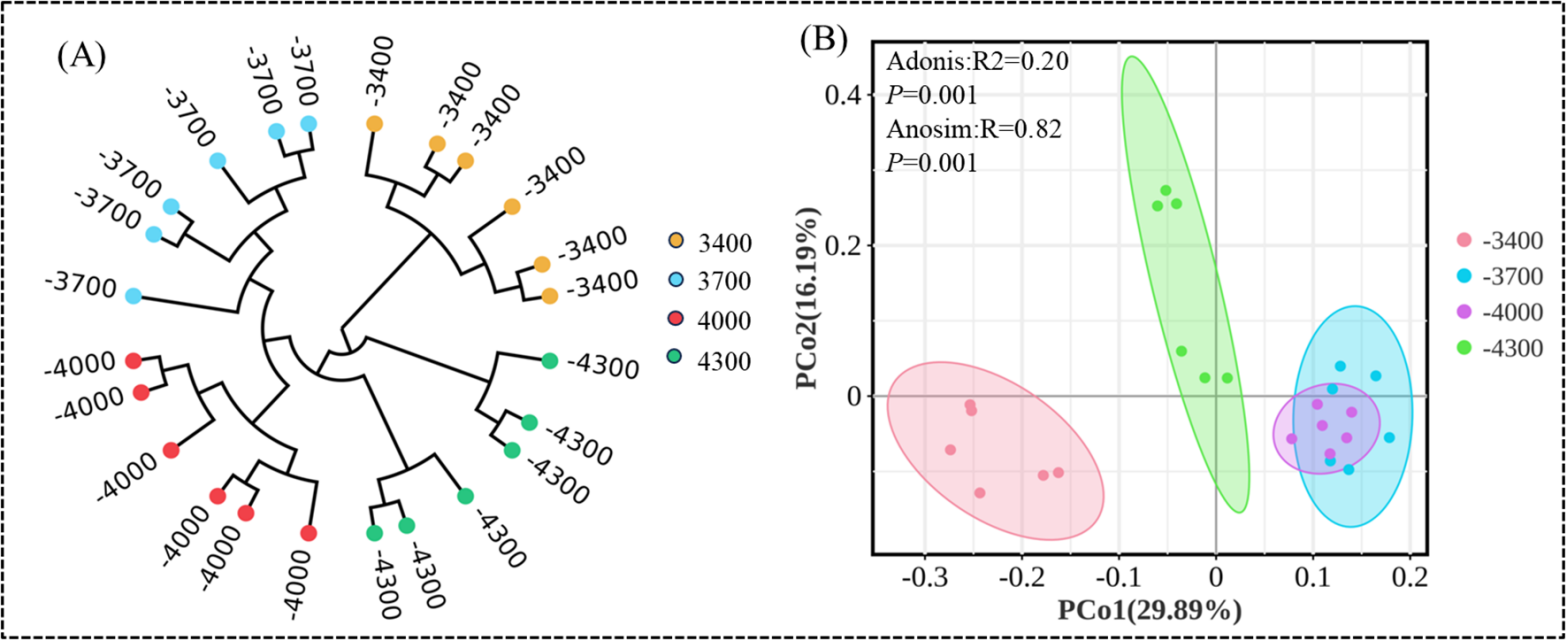
Arithmetic average unweighted paired grouping (UPGMA) clustering **(A)**, principal coordinate analysis of bacterial community diversity at different altitude gradients **(B)**.

UPGMA method and principal coordinate analysis (PCoA) were used to cluster soil bacterial communities (**Figure 5A**). UPGMA classification can study the similarity between different altitude gradients, and UPGMA identified four groups, namely 3700, 4000, 4300 and 3400 m, in which the community structure at 3700 and 4000 m is similar, while the community structure at 3400 m is different from other altitudes (**Figure 5B**). In addition, PcoA, ANOSIM and Adonis analysis showed that there were significant differences in bacterial communities among different altitude gradients (Adonis *P*=0.001; Anosim *P*=0.001), the first and second principal components explain 29.89% and 16.19% of the variation, respectively.

### 3.4 Indicator microbial groups of bacterial communities under different altitude gradients

**Figure 6:**
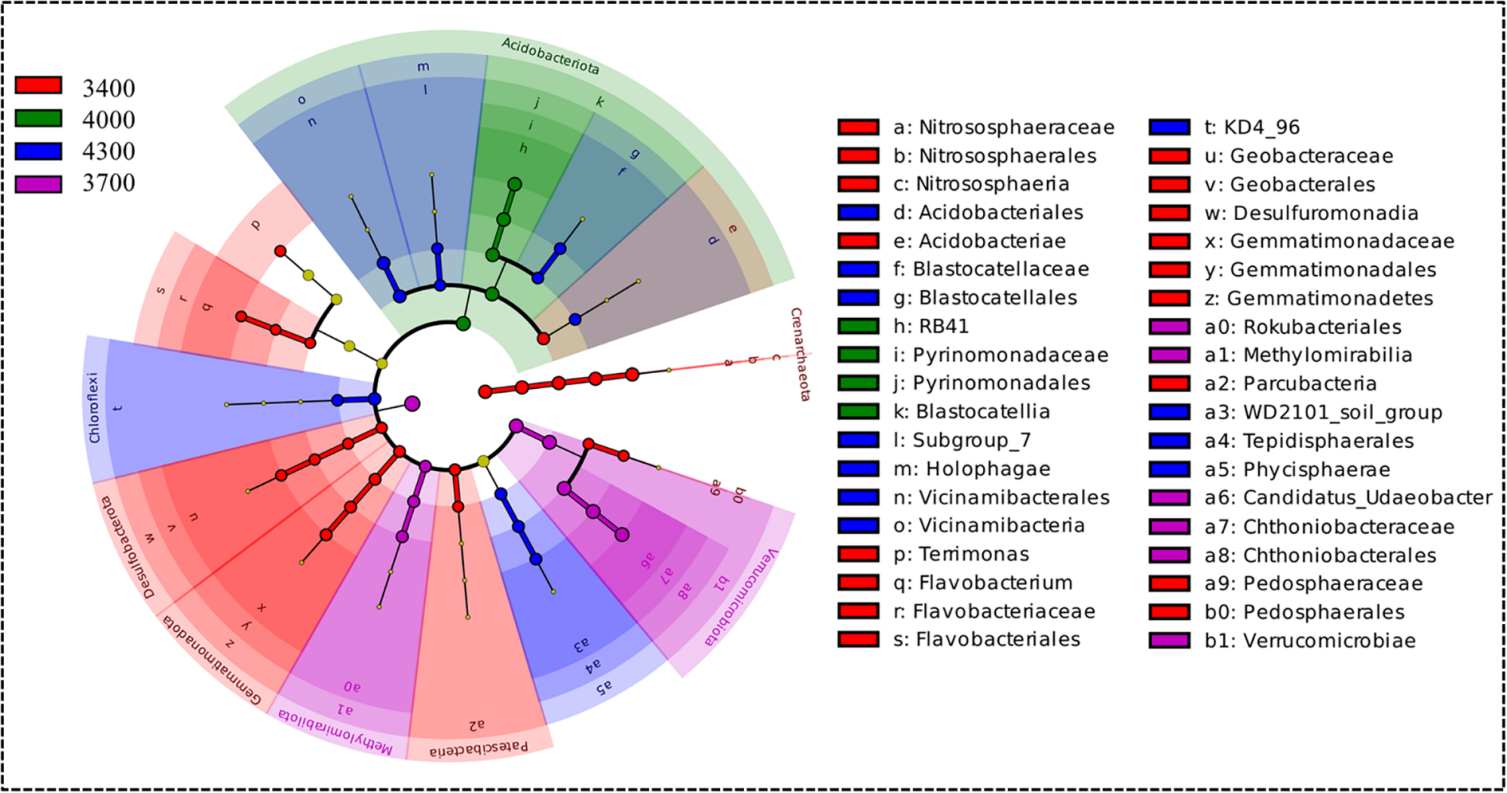
The phylogenetic tree diagram shows significant differences between bacterial enrichment groups, the LDA score diagram shows biomarkers at different altitudes, the groups with significant differences in abundance at different altitudes are represented by colored dots, and the phylogenetic groups from phylum to genus are represented by phylogenetic tree circles, only the LDA > 2.

LefSe analysis showed that altitude significantly changed the bacterial community. Specifically, at the level of phylum, class and genus, some bacterial groups are significantly enriched at different altitude gradients (**Figure 6**). The number of indicator bacteria at each altitude is as follows: 3400 (22), 3700 (9), 4000 (5) and 4300 (12) for 22 kinds of indicator bacteria determined by LDA>2.0. Most bacterial groups are mainly concentrated at an altitude of 3400 m. Specifically, at the phylum level, Crenarchaeota, Patescibacteria, Desulfobacterota and Gemmatimonadota are indicator bacteria at an altitude of 3400 m. Verrucomicrobiota and Methylomirabilota are indicator bacteria at an altitude of 3700 m. Acidobacteriota is an indicator bacterium at an altitude of 4000 m. Chloroflexi is an indicator bacterium at an altitude of 4300 m.

### 3.5 Changes of bacterial functional groups under different altitude gradients

**Figure 7:**
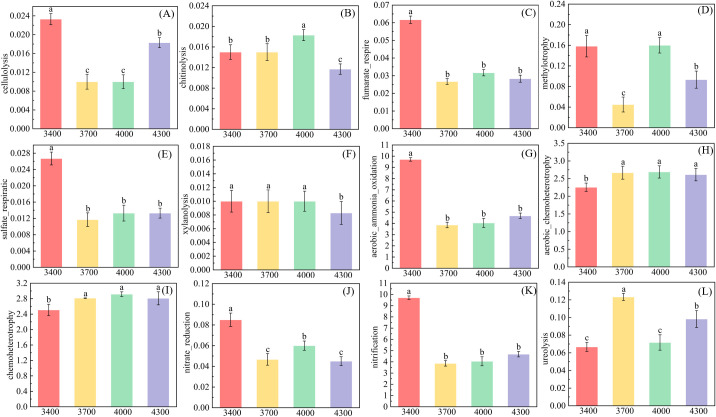
Changes of functional groups of soil bacteria at different altitudes. Different letters indicate significant differences between treatments (*P*< 0.05). **(A)** cellulolysis, **(B)** chitinolysis, **(C)** fumarate_respire, **(D)** methylotrophy, **(E)** sulfate_respiratic, **(F)** xylanolysis, **(G)** aerobic_ammonia_oxidation, **(H)** aerobic_chemoheterotrophy, **(I)** chemoheterotrophy, **(J)** nitrate reduction, **(K)** nitrification, **(L)** ureolysis.

Based on FAPROTAX database, the soil bacterial community was functionally labeled, and 56 functional groups were obtained. The results show that the altitude gradient significantly affects the functional groups related to carbon and nitrogen cycle (**Figure 7**). With the increase of altitude, functional groups related to nitrogen cycle, such as fumarate_respire, sulfate_respiratic and xylanolysis, showed a downward trend (**Figures 7C, E, F**), while celluloiysis first decreased and then increased (**Figure 7A**), and chitinolysis first increased and then decreased (**Figure 7B**). However, with the increase of altitude, mthylotrophy first decreases, then increases and then decreases, and the lowest is at 3700m (**Figure 7D**). Functional groups related to nitrogen cycle, tend to decrease with the increase of altitude (**Figures 7G, J, K**). However, the aerobic_chemoheterotrophy and Chemoheterotopy showed an upward trend with the elevation ladder (**Figures 7H, I**). Ureolysis first increases and then decreases with altitude, reaching the highest at 3700 m (**Figure 7L**).

### 3.6 The relationship between bacteria, soil, microbial biomass and enzyme activity

**Figure 8:**
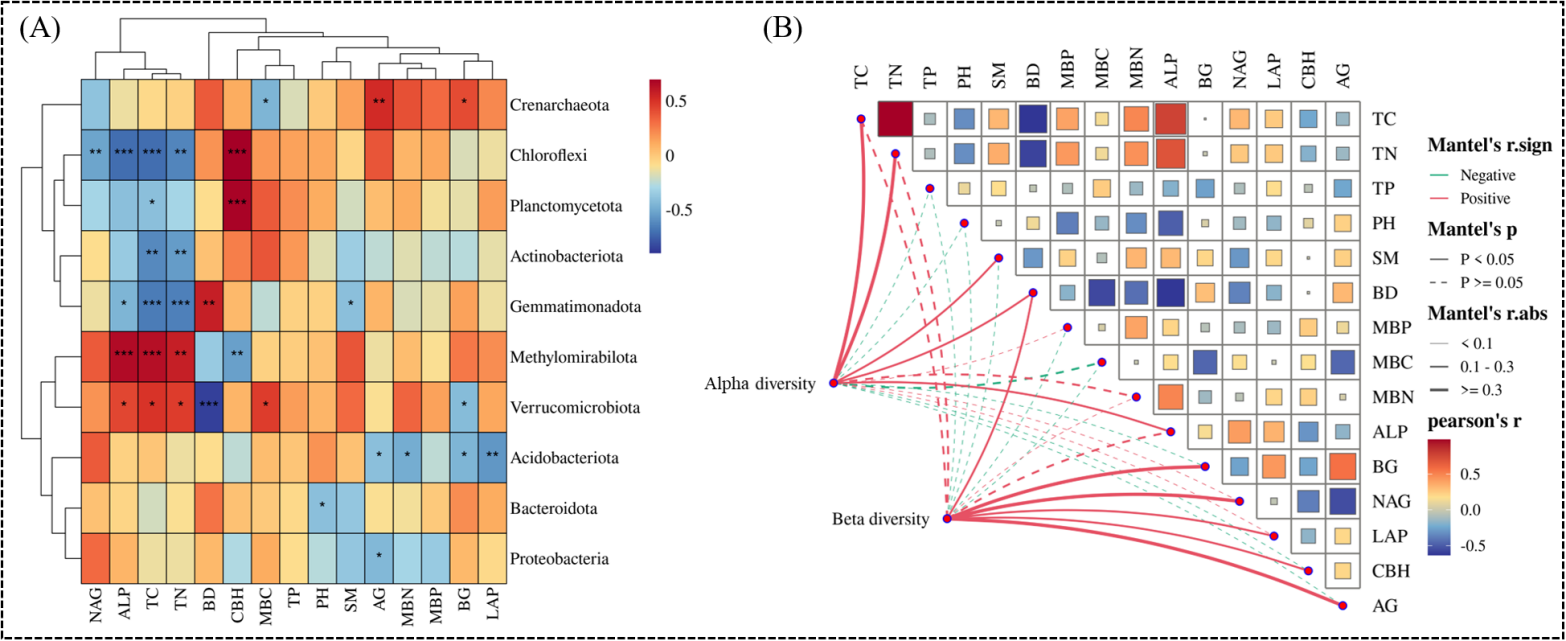
Thermal map of Spearman correlation coefficient between the top 10 dominant bacteria and soil factors **(A)**. Mental test of the relationship between α and β diversity and environmental factors **(B)**. *P<0.05,**P<0.01,***P<0.001.

Spearman correlation analysis showed that there was a significant relationship between dominant bacteria and soil factors (**Figure 8A**). NAG, ALP, TC and TN were negatively correlated with Chloroflexi. TC and TN were negatively correlated with Actinobacteriota and Gemmatimonadota. ALP,TC,TN and Methylomirabilota is a very significant positive correlation. There is a significant positive correlation between AG and Crenarchaeota (**Figure 8A**). Mantel test was used to evaluate the α diversity and β diversity of bacteria related to soil physical and chemical properties, microbial biomass and enzyme activity (**Figure 8B**). Mantel test revealed that the observed α diversity of bacteria was positively correlated with TC, TN, SM, BD and ALP. β diversity was positively correlated with BD, BG, NAG, LAP, CBH and AG (**Figure 8B**).

**Figure 9:**
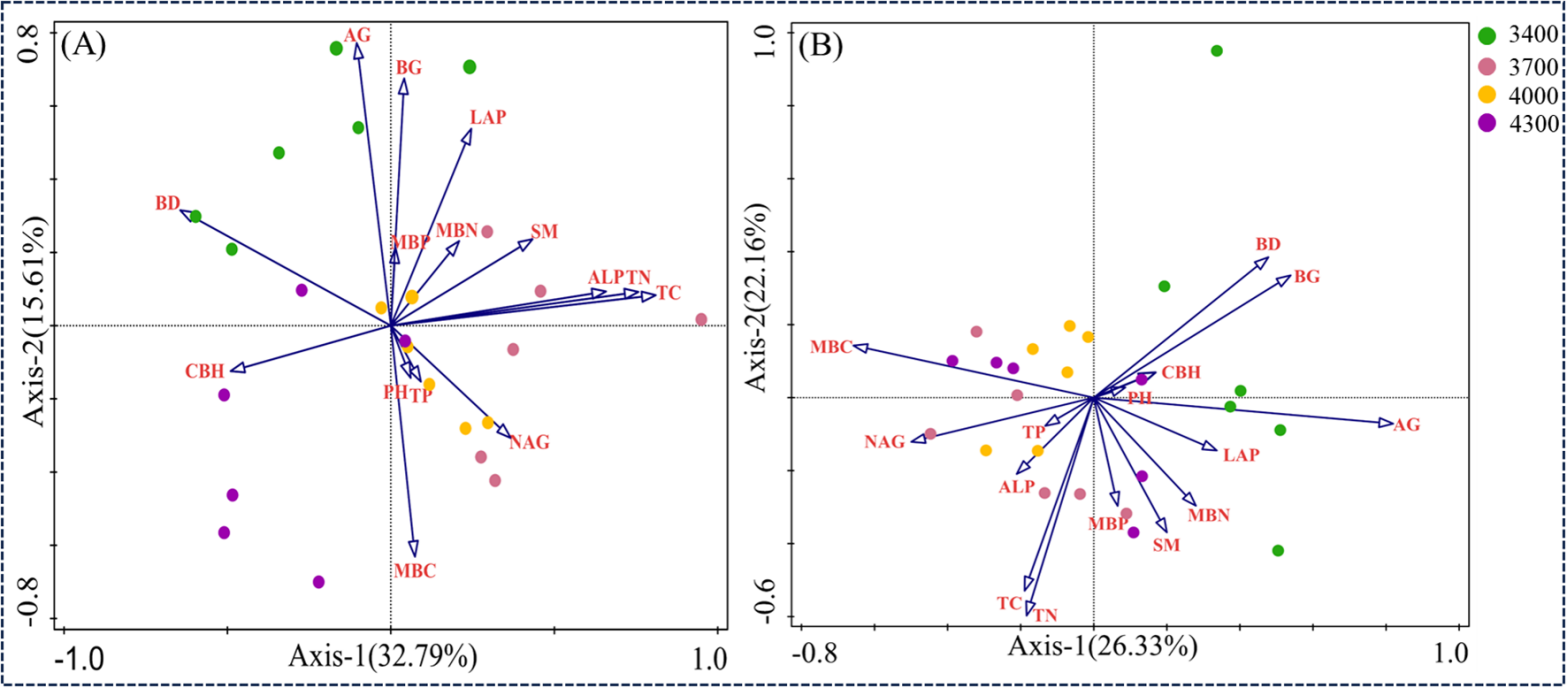
Ranking chart of redundant analysis (RDA) results to determine the relationship between bacterial communities and soil factors **(A)** and the relationship between bacterial functional groups and soil factors **(B)**.

RDA was used to analyze the relationship between soil bacterial communities and functional groups and soil nutrients and enzyme activities (**Figure 9**). In the first two axes, 32.79% and 15.61% were explained respectively (**Figure 9A**). The results showed that the main driving factors of soil bacterial community were TC, CBH, AG, BD and BG. The arrow of TC is the longest, which indicates that TC has the greatest influence on the overall distribution of bacterial communities in different altitude gradients, followed by CBH, AG, BD and BG. As shown in **Figure 8A**, the arrow of TC is the longest, which indicates that TC has the greatest influence on the overall distribution of bacterial communities in different altitude gradients, followed by CBH, AG, BD and BG. The results of Monte Carlo experiments show that the important values of soil physical and chemical properties decrease in the order of TC>CBH>Ag>BD>BG>pH>SM>LAP>MBC>MBP>NAG>TP>TN>MBN>ALP (**Table 2**). In addition, TC, CBH and AG are significantly different at the level of 0.01, while BD and BG are significantly different at the level of 0.05, and the contribution rates of these five factors are 22.3%, 11.3%, 11.4%, 6.6% and 5.8% respectively (**Table 2**). These results show that TC, CBH, AG, BD and BG are the main driving factors affecting the bacterial community structure at different altitude gradients. RDA was used to analyze the relationship between soil bacterial functional groups and soil nutrients and enzyme activities (**Figure 8**). In the first two axes, 22.33% and 22.16% were explained respectively (**Figure 9B**). The main factors affecting bacterial functional groups are AG, TN, BG, MBC and MBN. The difference of AG is significant at the level of 0.01, while that of TN, BG, MBC and MBN is significant at the level of 0.05. The contribution rates of these five factors are 18.60%, 8.8%, 7.0%, 7.0% and 6.6% respectively (**Table 3**).

**Table 2:** Characteristics of influencing factors on horizontal changes of soil bacteria phylum.

Soil physicochemical properties	Importance ranking	Explains (%)	Contribution	Pseudo-*F*	*P*
TC	1	22.3	29.1	6.3	0.002
CBH	2	11.3	14.7	3.6	0.004
AG	3	11.4	14.8	4.1	0.002
BD	4	6.6	8.6	2.6	0.054
BG	5	5.8	7.5	2.4	0.046
PH	6	4.8	6.2	2.2	0.1
SM	7	2.9	3.8	1.3	0.226
LAP	8	2.7	3.5	1.2	0.312
MBC	9	1.7	2.2	0.8	0.48
MBP	10	1.6	2.0	0.7	0.56
NAG	11	1.4	1.8	0.6	0.624
TP	12	0.9	1.1	0.4	0.826
TN	13	0.9	1.2	0.3	0.854
MBN	14	1.9	2.5	0.7	0.556
ALP	15	0.8	1.0	0.3	0.866

**Table 3:** Characteristics of influencing factors on changes of soil bacterial functional groups.

Soil physicochemical properties	Importance ranking	Explains (%)	Contribution	Pseudo-*F*	*P*
AG	1	18.6	26.0	5.0	0.002
TN	2	8.8	12.4	2.6	0.040
BG	3	7.0	9.8	2.1	0.048
MBC	4	7.0	9.8	2.3	0.028
MBN	5	6.6	9.2	2.3	0.042
BD	6	3.6	5.1	1.3	0.256
TC	7	3.4	4.7	1.2	0.320
TP	8	3.0	4.2	1.1	0.350
ALP	9	2.9	4.1	1.1	0.390
CBH	10	2.4	3.4	0.8	0.484
LAP	11	1.9	2.6	0.6	0.684
SM	12	2.1	2.9	0.7	0.616
PH	13	1.5	2.0	0.5	0.800
MBP	14	1.5	2.1	0.4	0.804
NAG	15	1.2	1.7	0.3	0.868

### 3.7 Co-occurrence network structure of different altitude gradients

Cluster analysis divides different altitude gradients into three groups (**Figure 3**), and the co-occurrence network reveals their significant differences (**Figure 6**). The complex relationship between nodes is described by calculating the topological properties of the network. Generally speaking, the number of 4300 m network nodes (191) is lower than 3400 (195), 3700 (198) and 4000 m (199) (**Figure 6A**), while the number of links is higher than 3700 (920) and 4000 m (1132) (**Figure 6B**). In addition, the altitude of 4300 m has a higher average degree and modularity (**Figures 6G, H**), indicating the cluster topology and modular structure. The network complexity at 4300 m is higher, indicating that the relationship between species is more complicated and the network stability is higher. On the contrary, the 3400 m network is more susceptible to environmental interference, with a modularity index of 0.53 (**Figure 6H**).

## 4 Discussion

### 4.1 Changes of soil physical and chemical properties, microbial biomass and extracellular enzymes in different altitude gradients

Altitude is one of the important topographical factors. Due to different altitudes, changes in climate characteristics and soil types all lead to differences in soil nutrients (Gilgen and Buchmann, 2009).Therefore, soil is affected by various ecological factors, and complex biological and abiotic factors closely interact to form a soil ecosystem (Denef et al., 2009). In this study, total carbon and total nitrogen first increased and then decreased with the elevation, which is consistent with the research conclusion of (Zhou, 2019). Altitude, as a natural geographical variation, is one of the important factors affecting the distribution of soil carbon and nitrogen (Xie L. L. et al., 2024). Due to altitude, continuous changes in heat, temperature, precipitation, and sunlight occur within the region (Bin et al., 2022). This is consistent with the findings of Gao et al. (2021). The total carbon (TC) and total nitrogen (TN) in the study area appeared at 3700 meters rather than 4300 meters. The main reason is that as the altitude rises to 3700m, human interference (grazing activities) gradually decreases compared to lower altitudes (3400m), soil moisture content gradually increases, and the content of plant litter and organic matter increases, which can provide more nutrients for the growth of soil microorganisms, promote the reproduction and activity of soil microorganisms, accelerate the utilization and mineralization rate of organic matter by soil microorganisms, thereby improving the physical and chemical properties of the soil (Pingree and Deluca, 2018). However, as the altitude continues to rise, wind erosion gradually worsens, the soil layer becomes thinner, the hydrothermal conditions deteriorate, and the shelter conditions decrease, causing significant changes in soil nutrients (Deng et al., 2019). Therefore, the total carbon and total nitrogen content of soil at different altitude gradients shows significant heterogeneity. Soil total phosphorus increased with the elevation (**Table 1**). A main driving factor seems to be that the degree of soil weathering decreases with the decrease of altitude, because soil phosphorus mainly comes from weathered soil (Helfenstein et al., 2018). Soil microbial biomass first increased and then decreased and increased with the elevation, and the microbial biomass was the highest at 3700 m above sea level (**Table 1**). The reason may be that the surface soil temperature at 3700 m above sea level is high, which is beneficial to plant growth and there are more exudates from vegetation roots, Higher soil temperature promoted microbial proliferation and increased microbial biomass (Uselman et al., 2000). It is worth noting that at 4300 m, microbial biomass phosphorus, microbial biomass carbon and microbial biomass nitrogen are higher than 4000 m, indicating that microbial biomass is affected by many factors (Ren et al., 2021). With the elevation, its climatic conditions will change (Xu et al., 2013). At the same time, soil microorganisms have a certain physiological adaptability to climate conditions. Although altitude increases will cause temperature drops, microorganisms can maintain balance by reducing metabolic activity (Li et al., 2014). Secondly, the low temperatures in high-altitude areas may increase physiological stress by altering osmotic pressure and reducing the ability to obtain effective nutrients Sun et al., 2025).

In this low-temperature environment, the decomposition of soil organic matter is slow, while the transformation of microbial biomass remains high (Wang B. R. et al., 2021; Wang R. et al., 2021). Soil extracellular enzymes can reveal the cycling law of nutrients such as C, N and P in soil, and reflect the intensity and direction of various biochemical processes (Kotroczo et al., 2014). At present, the most widely studied soil extracellular enzymes are BG,CBH,NAG,ALP and LAP, because they can be used as indicators of C demand, N demand and P demand respectively (Schimel and Weintraub, 2003). Specifically, BG hydrolyzes cellobiose into glucose during the carbon cycle, CBH hydrolyzes cellulose to produce sucrose during the carbon cycle, NAG hydrolyzes chitin during the carbon-nitrogen cycle, LAP hydrolyzes protein peptide during the nitrogen cycle, releasing amino acids from the N-terminal, and ALP hydrolyzes phosphate from phosphate sugars and phospholipids during the phosphorus cycle (Sinsabaugh et al., 2009). Previous studies have shown that altitude difference causes the change of micro-habitat, which has a gradient effect on soil physical and chemical properties, resulting in different soil enzyme activities (He et al., 2009). Studies such as Zhen et al. (2019) showed that soil enzyme activities showed significant differences between different altitudes. Our study found that the activities of ALP, BG, AG and LAP decreased significantly with the elevation, and our results were inconsistent with those recently reported, which reported that the soil enzyme activities in southern Patagonia did not respond significantly to the elevation gradient (Truong et al., 2019). This difference may be attributed to the different sampling points used in the two studies. In our study, the sampling points covered 3400-4300 m, while in the other study, the sampling points only covered 130-640 m. Different vegetation types in different regions respond differently to soil microbial communities and enzyme activities. In addition, In our research, we found that ALP is related to soil properties, especially to total carbon and total nitrogen content (S2). Our results are consistent with previous research reports that soil enzyme activities are strongly influenced by soil properties, especially total carbon (Truong et al., 2019). These results further confirmed that the extracellular enzyme activities associated with N and P cycling increased significantly with altitude, while those associated with C cycle decreased significantly with altitude, which supported our first hypothesis. To sum up, there is no unified conclusion on the influence of altitude gradient on soil enzyme activity, which is mainly due to the similarities and differences of regional and soil substrate conditions in the response of soil enzyme activity to microclimate change. When analyzing soil microbial activity, soil extracellular enzyme activity is also the most important analysis item. Therefore, the study on the dynamic relationship between soil extracellular enzyme activity and soil characteristics is helpful to understand the influence of microorganisms on ecosystem processes such as litter decomposition, soil carbon cycle and nutrient cycle.

### 4.2 Changes of bacterial community composition and structure at different altitude gradients

**Figure 10:**
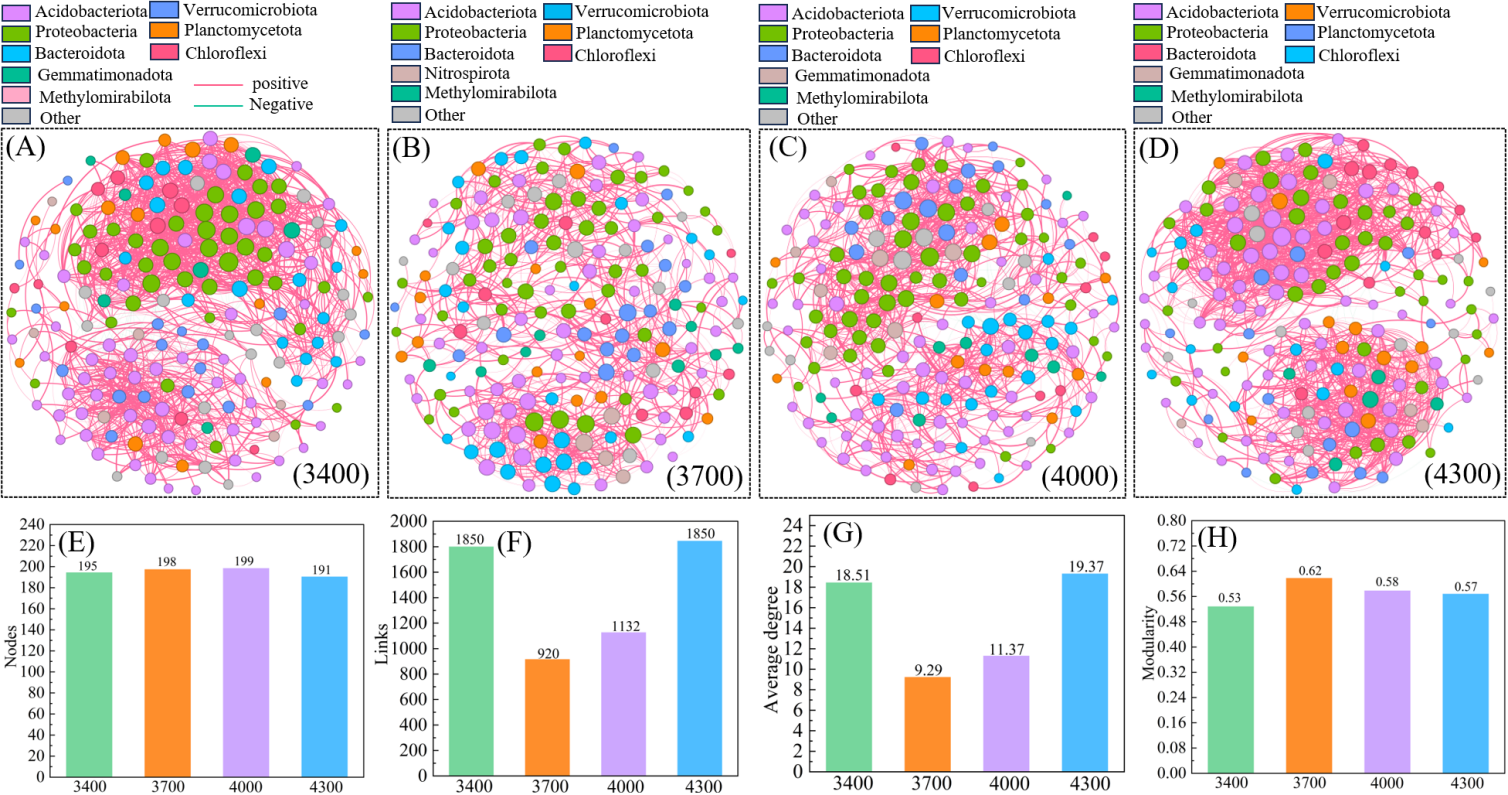
Based on correlation analysis, 3400 **(A)**, 3700 **(B)**, 4000 **(C)** and 4300 m **(D)** bacterial community coexistence networks. **(E–H)** represent the main characteristics of the co-occurrence network. The color and size of nodes represent the relative abundance of OTU groups and specific OTUs at the gate level.

Soil microorganisms are very sensitive to environmental changes, and macro variables such as altitude gradient cause changes in micro factors such as soil properties, temperature and moisture, thus affecting soil microbial community structure to varying degrees (Shen et al., 2013). In this study, UPGMA and principal coordinate (PCA) analysis found that bacterial communities at different altitudes can be well distinguished, and the characteristics of soil bacterial communities changed significantly with the change of altitude (**Figure 5**). The bacterial communities at 3400 m above sea level are obviously different from those at other altitudes. In addition, the bacterial communities at 3700 m, 4000 m and 4300 m above sea level are similar in composition and structure (Wang B. R. et al., 2021; Wang R. et al., 2021). These results show that different altitude gradients can lead to soil bacteria with different evolutionary characteristics. Specifically, in this study, Acidobacteria is the dominant bacteria, followed by Verrucomicrobiota and Proteobacteria. This is similar to Cui et al. (2019) finding that the dominant bacteria phylum is Actinomycetes, Bacteroides and Blastomycetes in the alpine ecosystem at an altitude of 2800-3500 m on the Qinghai-Tibet Plateau. Just as we found in our study that the relative abundance of various groups of microbial communities changed significantly with the elevation (**Figure 4A**), the co-occurrence model also supported our findings (**Figure 10**). Acidobacteria, Verrucomicrobiota and Proteobacteria are always the dominant populations of soil bacterial community at different altitudes, which have made great contributions to the variation of community composition, but the highest relative abundance of the three species appears at different altitudes. This result is similar to the bacterial diversity of Xie L. L. et al. (2024) in the desert habitat with an average elevation of 2800 m on the Qinghai-Tibet Plateau, in which Proteobacteria, Actinomycetes, Bacteroides and Chlorocurvata are the dominant groups, indicating that there are similar bacterial compositions in soils in different habitats, and at the same time, their respective dominant groups are similar.This is mainly related to the wide niche of Acidobacteria, Verrucomicrobiota and Proteobacteria, and their strong adaptability to different environments (Gao et al., 2019). In addition, the difference in adaptability of flora to microenvironment is the reason why the maximum relative abundance of the three major fungi appears at different altitudes. Studies have shown that Acidobacteria can improve soil nutrient content by degrading complex lignin and cellulose (Pankratov et al., 2011). Soil microbial communities respond to abiotic and biotic conditions. In mountain plant communities with vertical gradients, altitude can create unique microenvironments, which have unique microbial responses (Hernández-Cáceres et al., 2022). This shows that the distribution of soil bacteria is not only affected by altitude, but also by soil heterogeneity, which shows different research results. On the one hand, soil bacterial species have high spatial heterogeneity and are very sensitive to climate, soil and vegetation conditions, which leads to significant differences in dominant bacterial species in different research areas (Cao et al., 2022). This also shows the relative abundance of soil bacterial species in the high altitude area of Qinghai-Tibet Plateau, and the study of soil microorganisms still has great exploration space in this area. On the other hand, due to the existence of altitude gradient, the natural, soil and biological environment suddenly changed in a short geographical distance, and these gradients created a variety of bacterial ecological habitats, which led to great differences in soil bacteria species in the *Pfruticosa* shrub meadow under different altitude gradients on the Qinghai-Tibet Plateau (Hernández-Cáceres et al., 2022).

The α diversity of soil bacterial community directly reflects the change of soil bacterial community quantity and richness (Ding et al., 2023). Many studies show that α diversity decreases with the elevation (Yao et al., 2017). On the contrary, this study found that the diversity of soil bacteria in the study area was different at different altitude gradients (**Figure 3**), and the differences between different altitude gradients were obvious. At an altitude of 3400 m, there is the largest number of species unique to soil bacteria (**Figure 4B**). Studies have shown that specific soil microbial communities exist at specific altitudes (Liu et al., 2013). Shannon index and Pielou index of soil bacteria decreased first and then increased along the elevation gradient, and the maximum and minimum values appeared at the altitude of 3400 m and 3700 m respectively (**Figures 3A,C**). The reason may be related to the temperature and precipitation on the altitude gradient in Qinghai-Tibet Plateau (Gong et al., 2022). Therefore, the number and species of soil microorganisms decrease at the 3700 m. At the 3400 m, the bacterial diversity may increase slightly due to the increase of soil water content. In addition, the changes of soil Simpson index and good_coverage index along the elevation gradient are not obvious in this study (**Figures 3B, D**), which is consistent with the research results of Li et al. (2022) indicating that bacterial diversity may be difficult to form a unified pattern along the elevation gradient. These results further confirm our second hypothesis.

LEfSe analysis provides a new perspective for studying the response of soil bacteria to soil niche change.In the current research, most bacterial groups are obviously enriched at an altitude of 3400 m, indicating that these bacteria have a stable niche at an altitude of 3400 m. However, at an altitude of 3700 m, only Verrucomicrobiae is abundant. It is generally believed that in a long-term stable community (that is, a community with little interference), Some highly competitive species will reproduce, making a few species dominant in the community (Doležal et al., 2013).

The change of soil microbial community function is similar to the change of community structure, and different altitude gradients form different microbial functional groups. Different functional groups are often filtered into different environments, and these environments are the characteristics of biochemical cycles in these areas (Crowther et al., 2019). This study showed that at different altitudes, the bacterial functional group related to Nitrogen cycle changed obviously. Aerobics_ammonia_oxidation, nitrate_reduction and nitrification decrease with altitude, while aerobic_chemoheteotrophy, chemotetropy and ureolysis increase obviously with altitude. Aerobic_ammonia_oxidation and nitrification (**Figures 7G, K**) have the most obvious changes, which are much higher than other functions. This phenomenon and characteristics prove the protection strategy of limiting element nitrogen in different altitude gradients (Xi et al., 2017). It shows that the effect of nitrate_reduction is more intense at the altitude of 3400 m, and these different nitrogen-reducing bacteria groups may be related to the mechanism of soil nitrogen stability. For example, certain microbial communities (such as Bacillus, Pseudomonas and Bacillus subtilis) limit nitrogen loss by reducing nitrate to a more stable form (Kuypers et al., 2018). It is attributed to the fact that shrubs have nitrogen fixation characteristics (Hao et al., 2024). Furthermore, because Proteobacteria has a variety of species involved in nitrogen fixation, it can form a symbiotic relationship and fix nitrogen as a nitrogen compound that plants can use (Creamer et al., 2016). Similar to the change of microbial community structure, microbial functional groups at different altitudes are also different (**Figure 7**). The Carbon cycle of bacteria related to carbon cycle, such as cellulolysis, fumarate_respire, methylotrophy, sulfate_respiratic and xylanolysis, varies significantly with altitude, and their contents are all the highest at 3400 m. The relative abundance of methylotrophy is the largest, which may be due to the high vegetation coverage at 3400 m above sea level, and the great change of litter input will promote aerobic environment, thus accelerating the oxidation of CH4 (Qu et al., 2020).

### 4.3 Correlation between bacterial community and soil properties

The changes of soil microorganisms and functional groups in different altitude gradients of *P. fruticosa* shrub meadow are closely related to soil environmental factors (**Figure 9**). Spearman correlation shows that TC and TN are positively correlated with Methylomirabilota and Verrucomicrobilota (**Figure 9A**). TC is the most important factor affecting bacterial community and diversity (**Figure 8B**; **Table 1**). This is consistent with the research results of Jiang et al. (2021), which showed that microbial communities were significantly correlated with soil nutrients. The research shows that soil carbon accumulation affects the structure of soil microbial community under vegetation by affecting the diversity, productivity and stability of plant community, which explains why the change of TC will significantly affect the structure of bacterial community (Chen et al., 2021). This supports our second hypothesis. Secondly, the soil bulk density is the second environmental factor that affects the bacterial community, which may be because the rhizosphere soil of *P. fruticosa* shrub will produce the “ Fertility islands effect”, which makes the soil nutrient content enriched around its roots (Michalet et al., 2016). However, most microorganisms, animals and plants in the soil will draw enough nutrients for their own growth, so they concentrate on grabbing nutrients near the rhizosphere of the *P. fruticosa* shrub, which makes the soil nutrient content in short supply. However, due to the increase in altitude, the number of plants and animals decreases, and the exposed areas are relatively extensive, which makes the number of animals and plants in this area decrease obviously, and the number of soil microorganisms also decreases. Therefore, the dead bodies of litter and dead animals and plants cannot be effectively utilized, and the soil organic matter is low. It further aggravates the degree of soil hardening, resulting in high soil bulk density, poor water stability and serious soil water loss. The local micro-habitat situation is not conducive to the reproduction and metabolism of soil microorganisms, so soil bulk density has gradually become the main environmental limiting factor to limit bacteria (Yang et al., 2013). Soil extracellular enzymes are closely related to a series of biological and abiotic factors (Ivashchenko et al., 2021). In our study, TC and TN were positively correlated with ALP (S2), and CBH and AG were positively correlated with Chloroflexi, Planctomycetota and Crenarchaeota (**Figure 8A**). This confirms the view that soil total nutrients play a key role in the altitude model of regulating extracellular enzyme activity (Cao et al., 2021).

The main environmental limiting factor in bacterial functional groups is total nitrogen (**Figures 9B**, **Table 2**). This may be because different microbial functional groups perform different functions at different stages of the material cycle, and they work together to promote the rapid and orderly flow of matter and energy in the ecosystem and ensure the normal succession of grassland ecosystem (Gordon et al., 2008). The change of environmental factors has a direct impact on microbial functional groups. Nitrogen-fixing bacteria transform N_2_ into NH4^+^ for plants to synthesize organic nitrogen and supplement nitrogen in grassland soil; Ammoniated bacteria nitrated NH4^+^ into NO3^-^; Nitrifying bacteria convert NO3^-^ into N_2_ to complete the nitrogen cycle, which is related to the enzyme activity, so soil enzyme activity is also the main factor affecting microbial functional groups (Wang B. R. et al., 2021; Wang R. et al., 2021). Nevertheless, due to the large reduction of plants and microorganisms and the intensification of soil erosion caused by bare plots, the only soil nutrients are still not enough to maintain the normal growth of plants and microorganisms, and the competition among species is intensified (Ahmed et al., 2019). Soil nitrogen content is an indispensable environmental factor to maintain the normal flow of microbial functional groups, so soil total nitrogen is the main environmental factor to limit microbial functional groups (Yang et al., 2008). Secondly, the influencing factors are microbial biomass carbon, nitrogen and extracellular enzymes (AG and BG) related to carbon cycle. Microbial biomass carbon is an important indicator reflecting the size of microbial community, and extracellular enzymes are mainly produced by microorganisms, and the size of microbial biomass determines the potential of enzyme production (De Vries et al., 2018). Soil microbial biomass carbon is an important part of active organic carbon pool. Because of its fast turnover rate and easy decomposition, it is also a great supply source of soil effective nutrient pool (Wang B. R. et al., 2021; Wang R. et al., 2021 ). These fast-moving carbon sources can continuously provide energy for soil microorganisms and keep them relatively high in activity, so as to drive the biogeochemical cycle of nutrients such as carbon and nitrogen, and then affect microbial functional groups (Zhou et al., 2022).

### 4.4 Co-occurrence patterns of bacteria under different altitude gradients

Species form a complex network system by secreting metabolites and interacting with other microorganisms (Peura et al., 2015). The co-occurrence network among microorganisms reflects the function and stability of biological communities (Ma et al., 2020). It provides a preliminary means to explore the organization and dynamics of microbial interaction and niche (Banerjee et al., 2019). It is widely used to estimate co-occurrence patterns in complex microbial relationships (Deng et al., 2012). It has been pointed out that the positive correlation connection in the co-occurrence network represents the mutual synergy between microorganisms, while the negative correlation connection represents the potential antagonism between microorganisms (Blanchet et al., 2020). In this study, the connection of soil bacterial co-occurrence network is mainly positive correlation (bacterial network: 97.26%-99.3% positive correlation link), and the complexity of soil microbial network is the highest at 4300 m, followed by 3400 and 4000 m, and the complexity of 3700 m is the lowest (**Figure 10**). These differences are reflected in factors such as average degree and modularity index. Our research is consistent with Duan et al. (2021) research on microbial co-occurrence network in Qinghai-Tibet Plateau. This may indicate that when microbial communities are stressed by cold and harsh environment (such as strong solar radiation, low soil temperature and low soil oxygen content), soil microbial communities can maintain their ability to resist external interference through mutual cooperation (Hernández-Cáceres et al., 2022). The complexity of the co-occurrence network tends to increase with the altitude, which indicates that the scale of the microbial co-occurrence network is large in high altitude areas, and the interaction between microorganisms has been enhanced. Similar conclusions have been drawn in the previous research on the bacterial co-occurrence network in Gongga Mountain (Zhu et al., 2020). The possible reason is that the microbial community has been stressed by the cold and harsh environment (Hernández-Cáceres et al., 2022). This shows that the soil microbial community in high altitude areas is under greater environmental stress than that in low altitude areas, and the co-occurrence network structure of soil microorganisms in high altitude areas will become more complicated. Therefore, more diverse and richer microbial groups create greater possibilities, that is, there may be more potential taxa in the environment to participate in potential interactions, thus leading to more complex association networks.

## 5 Conclusion

This study explored the distribution patterns and driving factors of soil bacterial community structure, diversity, and enzyme activity along an altitude gradient in *P*. fruticosa shrub meadows. It was found that the soil bacteria in *P. fruticosa* shrubs have different adaptabilities, which are closely related to soil nutrients and enzyme activity. This study provides a new perspective for further understanding the distribution pattern and driving forces of soil bacterial communities in *P. fruticosa* shrubs along the altitude gradient, enhancing our understanding of microbial ecology in alpine shrub environments. We observed significant differences in soil bacterial α-diversity and community composition at different altitudinal gradients. The altitudinal gradient influences soil bacterial communities by correlating with soil properties, microbial biomass, and enzyme activity. Soil properties (TC and BD) and enzyme activities (CBH, AG, and BG) are the main factors affecting changes in soil bacterial communities, while AG, TN, BG, MBC, and MBN have significant impacts on bacterial functional groups.

We also found that TC, TN, SM, MBC, MBN, and MBP were highest at 3700 m altitude, while ALP was the most active. Moreover, the dominant bacterial phylum shifted from Acidobacteria (at 3400 m) to Verrucomicrobia (at 3700 m). This shows that this area has special ecological adaptability and should be protected first to avoid interference. It is recommended that protective land use measures (such as restricting overgrazing and development) be implemented in this area, and a monitoring system for *P. fruticosa* shrub vegetation be established to keep track of its growth and water conservation status in real time. Based on the monitoring data, protection and management measures should be adjusted in a timely manner to maintain its unique microbial community and ecological functions, thereby ensuring the long-term stability and sustainable development of the ecosystem.

## Data Availability

The original contributions presented in the study are included in the article/supplementary material. Further inquiries can be directed to the corresponding authors.
