# Supplementary material for: Changes of soil bacterial community composition and functional groups in different altitude gradients of Potentilla fruticosa shrub in eastern Qinghai-Tibet Plateau
Source: Front Plant Sci. 2025 Oct 20;16:1539945. doi: 10.3389/fpls.2025.1539945 (PMC12580278; doi:10.3389/fpls.2025.1539945)
Supplement: Supplementary file 1 [file DataSheet1.docx]

Supplementary Material


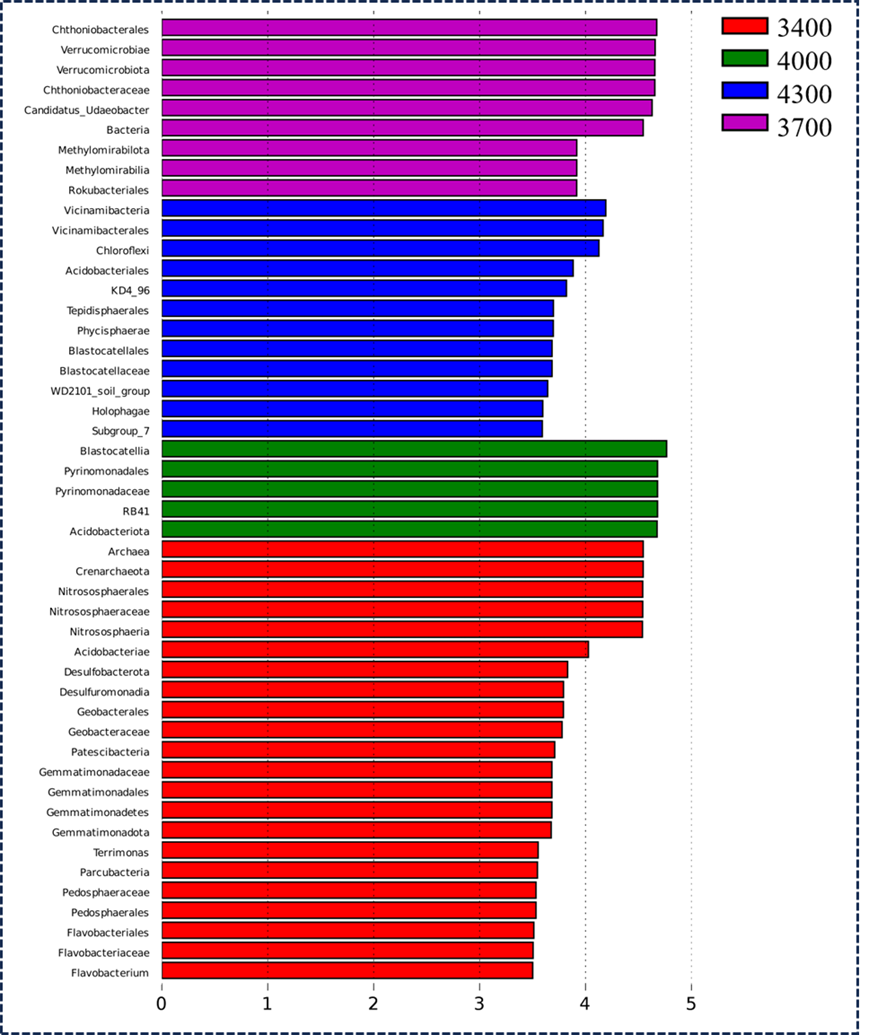


Figure S1. The score chart shows the LDA scores of biomarkers and bacterial taxa with significantly different abundance at different altitudes.


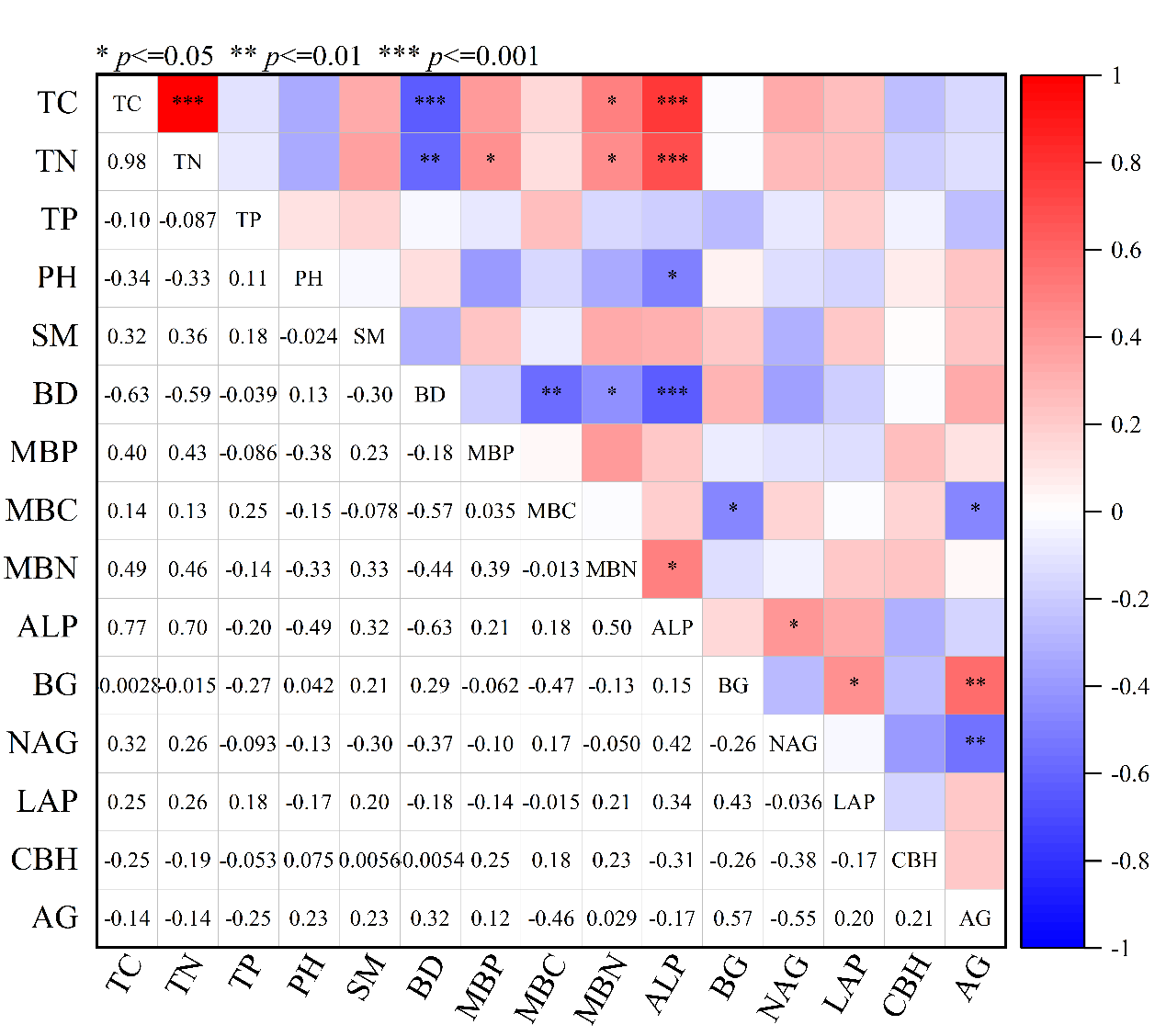


Figure S2. Correlation analysis of soil properties at different altitude gradients.
